# Genetic diversity of *Listeria monocytogenes* from seafood products, its processing environment, and clinical origin in the Western Cape, South Africa using whole genome sequencing

**DOI:** 10.3934/microbiol.2024029

**Published:** 2024-08-07

**Authors:** Karlene Lambrechts, Pieter Gouws, Diane Rip

**Affiliations:** Department of Food Science, Stellenbosch University, 7602, South Africa

**Keywords:** *Listeria monocytogenes*, seafood, fish, clinical, whole genome sequencing, South Africa

## Abstract

*Listeria monocytogenes* is a concern in seafood and its food processing environment (FPE). Several outbreaks globally have been linked to various types of seafood. Genetic profiling of *L. monocytogenes* is valuable to track bacterial contamination throughout the FPE and in understanding persistence mechanisms, with limited studies from South Africa. Forty-six *L. monocytogenes* isolates from origins: Fish/seafood products (n = 32) (salmon, smoked trout, fresh hake, oysters), the FPE (n = 6), and clinical (n = 8) were included in this study. Lineage typing, antibiotic susceptibility testing, and screening for two genes (*bcrABC* and *emrC*) conferring sanitizer tolerance was conducted. The seafood and FPE isolates originated from seven different factories processing various seafood products with undetermined origin. All clinical isolates were categorized as lineage I, and seafood and FPE isolates were mostly categorized into lineage II (p < 0.01). Seafood and FPE isolates (53%) carried the *bcrABC* gene cassette and one fish isolate, the *emrC* gene. A subset, n = 24, was grouped into serotypes, sequence types (STs), and clonal complexes (CCs) with whole genome sequencing (WGS). Eight CCs and ten STs were identified. All clinical isolates belonged to serogroup 4b, hypervirulent CC1. CC121 was the most prevalent in isolates from food and the FPE. All isolates carried *Listeria* pathogenicity islands (LIPI) 1 and 2. LIPI-3 and LIPI-4 were found in certain isolates. We identified genetic determinants linked to enhanced survival in the FPE, including stress survival islets (SSI) and genes conferring tolerance to sanitizers. SSI-1 was found in 44% isolates from seafood and the FPE. SSI-2 was found in all the ST121 seafood isolates. Isolates (42%) harbored transposon Tn1688_qac (*ermC*), conferring tolerance to quaternary ammonium compounds. Five plasmids were identified in 13 isolates from seafood and the FPE. This is the first One Health study reporting on *L. monocytogenes* genetic diversity, virulence and resistance profiles from various types of seafood and its FPE in South Africa.

## 1. Introduction

*Listeria monocytogenes* is a Gram-positive, non-spore forming, rod-shaped, facultative anaerobic human pathogen that causes listeriosis, which is mainly transmitted through contaminated food, affecting public health worldwide. *L. monocytogenes* is commonly found in environmental sources like soil, plant material, silage, fresh and salt water, faeces of healthy animals, and raw foods associated with these environments [1]–[4]. Furthermore, it is also commonly isolated from the food processing environment (FPE) and retail outlets. *L. monocytogenes* can grow and adapt to a range of different environmental stress conditions, including a range of pH conditions, temperatures (1 °C to 50 °C), high salt concentrations, low water activity, and low nutrient availability [5]–[7]. These adaptive characteristics of *L. monocytogenes* to diverse environments make this pathogen a great concern where ready-to-eat (RTE) foods are consumed without heating, or cooking [6],[8].

*L. monocytogenes* can be categorized into four genetic lineages (I-IV) with each lineage comprised of distinct serotypes [7],[9]. Distinction between the lineages are based on the variation within the nucleotide sequence of the virulence genes and genetic differences associated with the cell wall [10],[11]. The different lineages have different host tropisms and are associated with different levels of pathogenicity towards humans [12]. Lineage I is commonly associated with human listeriosis whereas lineage II is frequently isolated from food and the FPE [11],[13],[14]. Lineages III and IV are rarely isolated from food or the FPE and occur mostly in ruminants, being less pathogenic compared to lineages I and II [9],[10],[13].

Mortality rates of listerioses varies between 20–30% with higher rates in immunocompromised and immunocompetent individuals [15],[16]. Given the high listeriosis fatality rate (13% in 2020) [17] and variation in antibiotic resistance patterns across different countries, it is important to continuously screen and assess the efficiency of antibiotics prescribed [18]. Antibiotic resistance is of major concern and increasingly reported in numerous studies worldwide [18]–[22]. Several antibiotics have been used to treat listeriosis in patients. Ampicillin or penicillin G in combination with an aminoglycoside (like gentamycin), trimethoprim in combination with a sulfonamide, tetracycline, erythromycin, and vancomycin are all antibiotics that can be used to treat listeriosis [9],[18],[21]–[23].

*L. monocytogenes* is known to be present and persist in the FPE, and cleaning, sanitation, and hygiene practises are ways to control it. *L. monocytogenes* is increasingly showing tolerance to benzalkonium chloride (BC), a quaternary ammonium compound (QAC), as well as other QACs commonly used in the FPE to control *L. monocytogenes* and other microorganisms [24]–[26]. BCs are also commonly used in retail, households, personal care use, and the clinical setting [27],[28]. A number of genes (*bcrABC* cassette, *emrC, emrE, qacA, qacC and qacH*) associated with tolerance to QACs, based on the efflux pump system, have been identified in *Listeria* species [24],[29]. These genes enhance the ability of *L. monocytogenes* to survive higher concentrations of QAC sanitizers by actively pumping it out of the cell [26],[29]–[32]. These genes and subsequent effects make it more difficult to get rid of persistent strains in the FPE and is of growing concern.

A *Listeria* pathogenicity island (LIPI) is a well-defined gene cluster in the chromosome of the microorganism playing important roles in the virulence of *L. monocytogenes* [8],[33]. LIPI-1 and LIPI-2 regulates the pathogenicity and virulence of *L. monocytogenes* [19]. LIPI-3 consists of a listeriolysin S (LLS), a biosynthetic cluster of eight genes enhancing invasion [5]. It produces a haemolytic and cytotoxic factor important in the infection of murine animals and aids in the survival of polymorphonuclear cells [34]. This pathogenicity island is linked to the increased virulence potential of *L. monocytogenes*. The alternations it causes in the host microbiota during infection, facilitate intestinal colonisation [5],[35]. LLS is expressed under oxidative stress conditions conferring a better ability to escape the phagosome, thereby increasing virulence [36]. LIPI-4, containing six genes, encodes for a cellobiose-family phosphotransferase system found in hypervirulent clonal complexes (CCs). It is mostly found in lineage I isolates and is responsible for enhanced cell invasion, central nervous system tropism and placental invasion, which poses elevated risk for pregnant woman [33],[36]–[38].

SSI-1 is a five-gene stress survival islet (*lmo0444, lmo0445, lmo0446 (pva), lmo0447 (gadD1)* and *lmo0448 (gadT1)*), and is suggested to help the bacterium grow better under acidic, osmotic, gastric, and bile stresses, and high salt concentration [32],[38]–[41]. The growth advantage of *L. monocytogenes* in low pH environments are important, allowing it to survive the acidic environment encountered in the gastrointestinal tract [42]. This advantage to early gastrointestinal survival [41], can increase the possibility of *L. monocytogenes* enduring the stomach environment and crossing the intestinal membrane, leading to infection. The stress survival islet, SSI-2, consists of two genes-the transcription factor gene (*lin0464*) and an intracellular *pfpl* protease gene (*lin0465*) [40],[42]. It is also known to enhance *L. monocytogenes'* survival in food and provide advantages in the FPE [39],[41],[43]. SSI-2 has shown to provide a growth advantage under alkaline and oxidative stress conditions relevant in a FPE [32],[39],[40].

Whole genome sequencing (WGS) is increasingly used in the food industry for proactive surveillance and identifying the possible source of contamination [5]. WGS data allows for in-depth genetic characterization of the pathogen and information such as virulence potential, antimicrobial resistance and stress tolerance is provided. Serotyping is a typing method based on the presence of somatic (O) and flagellar (H) antigens on the complex and variable outer surface of the bacterial cell [6],[44]–[46]. *L. monocytogenes* can then be further categorized into different sequence types (STs) based on the seven-housekeeping genes and depending on the variation of the nucleotide sequences of these genes, a ST is assigned [47]–[49]. STs can be grouped together in CCs, where every ST in that group shares at least five of the seven identical alleles with one other ST in that group [47]–[49]. Certain STs and CCs are more prevalent and may be associated with higher occurrences of clinical cases, or more commonly found in food and the FPE compared to others. However, all *L. monocytogenes* STs are considered pathogenic and considered to carry the same risk and relevance to food safety by regulatory authorities [5].

We adopted a One Health approach by examining *L. monocytogenes* isolates from clinical, food, and environmental sources. Very little is known about the resistance and virulence profile of *L. monocytogenes* from seafood origin and its FPE in the Western Cape, South Africa. By comparing isolates from various origins in terms of genetic determinants, we aim to understand the mechanisms these isolates use to counteract stresses in food production environments and the human host. Understanding how this pathogen behaves in these interconnected ecosystems is beneficial for risk assessment. Our objectives were to classify forty-six (n = 46) *L. monocytogenes* isolates from seafood (salmon, trout, hake, oysters etc.), FPE (seven different factories), and clinical origin into lineage groups. It also screened for the presence of two genes associated with sanitizer tolerance, namely *emrC* (efflux-mediated resistance gene C) and *bcrABC* (benzalkonium chloride resistance cassette), using PCR, and conducted phenotypic antibiotic susceptibility testing against seven clinically relevant antibiotics. Lineage classification, together with the factory origin, date of isolation and product types were used to discuss possible sources of contamination. The outputs informed our selection of 24 isolates for WGS to determine virulence and resistance factors.

## 2. Materials and methods

### 2.1. Sample collection and storage

A total of forty-six (n = 46) isolates were investigated in this study, collected from various origins (food, environment and clinical). Thirty-eight of the isolates from seafood products (n = 32) and the FPE (n = 6) were distinguished from other food isolates stored in the culture bank (Department of Food Science, Stellenbosch University) over several years (2018–2022). Food and FPE isolates were received from Microchem Lab Services (Pty) Ltd, an independent, SANAS (South African National Accreditation System) accredited testing laboratory in the Western Cape, South Africa. These *L. monocytogenes* isolates were received on RAPID'L.Mono™ Chromogenic Media (Bio-Rad), purified on RAPID'L.Mono™ and 2% blood agar (National Health Laboratory Services (NHLS), Greenpoint) and stored in skim milk, tryptone, glucose and glycerine medium (STGG) (NHLS, Greenpoint). These were stored at −20 °C as glycerol stocks. The seafood and FPE isolates originated from seven different food establishments (Factories-A through -G) in the Western Cape where processing and packaging of seafood products take place. The FPE isolates (n = 6) included fish room floor (n = 1), drains (n = 4) and drain offcuts (n = 1). The seafood products included: salmon and salmon products (n = 11), trout and trout products (n = 12), and hake, tuna, oysters, seabass fillets, fish (n = 9). Upon inquiry, the origin of the salmon was unknown. Samples were received from local suppliers/distributors.

Eight clinical isolates (n = 8), from patients with listeriosis, from 2019, were obtained from the NHLS, sub-cultured onto RAPID'L.Mono™ and 2% blood agar (NHLS) for purity, and stored in STGG medium (NHLS) at −20 °C.

### 2.2. Phenotypic testing for Listeria monocytogenes

Stock cultures (stored in STGG at −20 °C) were streaked on RAPID'L.Mono™ Chromogenic Media (Bio-Rad) and incubated at 37 °C for 24 h. One presumptive *L. monocytogenes* colony (blue colony) from RAPID'L.Mono™ Chromogenic Media was then re-streaked with a sterile inoculation loop (Lasec SA) on 2% blood agar (NHLS, Greenpoint), for purity, and incubated at 37 °C for 24–48 h. Pure *L. monocytogenes* colonies from the blood agar were used for DNA extractions (below) and preserved at −20 °C in STGG medium for further analysis.

### 2.3. Deoxyribonucleic acid (DNA) extraction

DNA extractions were performed from 2% blood agar using a Quick-DNA™ Fungal/Bacterial Miniprep Kit (ZymoResearch) according to manufacturer's instructions. The extracted DNA was stored at −20 °C for further analysis. A negative DNA extraction control (where *L. monocytogenes* culture was absent in the DNA extraction) was also prepared.

### 2.4. Screening for the hly gene by PCR

The method developed by authors [50] and subsequently optimized [11] was used to confirm phenotypic presumptive positives of *L. monocytogenes* by screening for the hemolysin (*hly*) gene. DNA extracted from *L. monocytogenes* was applied as template for PCR assays. The T100 Thermal Cycler (Bio-Rad, South Africa) was used for PCR amplifications.

Each 25 µL PCR mixture contained nuclease-free water, 1 X final concentration NH4 reaction buffer (Bioline, Celtic Molecular Diagnostics), 0.2 mM final concentration deoxynucleoside triphosphate mix (ThermoFischer Scientific), 3 mM final concentration MgCl_2_ solution (Bioline), 0.3 µM final concentrations of each primer (forward and reverse) (Whitehead Scientific) [50], 1 U of BioTaq DNA polymerase (Bioline) and 1 µL of the target DNA. The PCR cycling conditions consisted of an initial denaturation at 94 °C for 3 min, followed by 30 cycles of denaturation at 94 °C for 40 s, annealing at 55 °C for 40 s and extension at 72 °C for 40 s with a final extension at 72 °C for 5 min. DNA from *L. monocytogenes* ATCC 23074 (serotype 4b) was used as a positive PCR control, and Molecular Biology Grade Water (HyClone™) was used as a negative PCR control. The negative DNA extraction control was included in the assay.

Amplified PCR products were separated by electrophoresis using 1.5% agarose gel (Lonza, Whitehead Scientific) in 1 X TAE buffer supplemented with smart glow pre-stain (0.05 µL/mL) (Accuris, Whitehead Scientific). A 100 bp DNA ladder (ThermoFischer Scientific) was incorporated in each gel run. Electrophoresis was conducted at 90 Volt for 90–120 min and images were captured using a Gel Doc™ XR+ instrument (Bio-Rad, South Africa) with Image Lab™ Software.

### 2.5. Lineage typing with PCR-RFLP

*L. monocytogenes* isolates were categorised into lineage groups (I, II or III) using a single nucleotide polymorphism-restriction fragment length polymorphism (SNP-RFLP) method on the *hly* gene amplicon [11].

### 2.6. Phenotypic antibiotic susceptibility testing

Phenotypic antimicrobial susceptibility was determined using the disc diffusion method [51]. A total of seven antibiotics were used with the concentrations listed in Table 1: Ampicillin, erythromycin, gentamicin, tetracycline, meropenem, co-trimoxazole, and chloramphenicol (Oxoid, ThermoFischer Scientific). The choice of antibiotics tested was based on the treatment options for listeriosis in South Africa.

*L. monocytogenes* isolates were streaked from STGG stock cultures onto RAPID'L.Mono™ Chromogenic Media (Bio-Rad) with a sterile inoculation loop (Lasec) and incubated at 37 °C for 24 h. One presumptive *L. monocytogenes* (blue colony) was then re-streaked on 2% blood agar (NHLS), for purity, and incubated at 37 °C for 24 h. *L. monocytogenes* colonies were picked up and suspended into 3 mL sterile saline solution (0.45% NaCl) (bioMérieux, South Africa) to achieve a McFarland standard of 0.5 using the VITEK® DENSICHEK® (bioMérieux, South Africa). This instrument was only used to measure the optical density of the microbial suspension. Within thirty minutes of making the bacterial cell suspension, it was spread on Mueller-Hinton agar supplemented with 5% defibrinated horse blood and 20 mg/L β-NAD (MH-F) (ThermoFischer Scientific) using a sterile cotton swab. The antimicrobial discs were placed on the agar (maximum of 4 discs on one plate) and incubated for 24 h at 37 °C. Each sample was tested in duplicate. The diameter of the zones of inhibition (Table 1) were measured (mm) around the discs after 24 h.

Minimum inhibitory concentration (MIC) breakpoints were assessed based on the European Committee on Antimicrobial Susceptibility Testing (EUCAST) [52]–[53]. In the case of chloramphenicol, gentamycin, and tetracycline where no breakpoint values were available for *L. monocytogenes*, the breakpoint values of *Staphylococcus* spp. were used [54]–[56]. *Staphylococcus aureus* ATCC 25923 (Davies Diagnostics) was used as a control strain [56],[57]. An isolate would be classified multidrug-resistant (MDR) when it shows resistance to at least one antimicrobial agent in three or more antimicrobial classes [52].

**Table 1.** Antibiotics with disc concentration and breakpoint values [53].

Antibiotic	Class	Disc content (µg)	Zone diameter breakpoints (mm)	
			Susceptible	Resistant
Ampicillin (AMP)	Penicillin	2	≥16	<16
Erythromycin ®	Macrolides	15	≥25	<25
Gentamicin (CN)	Aminoglycosides	10	≥18	<18
Tetracycline (TE)	Tetracyclines	30	≥22	<19
Meropenem (MEM)	Carbapenems	10	≥26	<26
Trimethoprim/Sulphamethoxazole (SXT)	Sulfonamides	1.25–23.75	≥29	<29
Chloramphenicol ®	Phenicols	30	≥18	<18

### 2.7. Screening for genes contributing to sanitizer tolerance

DNA extracted from *L. monocytogenes* was applied as template for PCR assays. PCR primer sequences and amplicon sizes for the *emrC* and *bcrABC* genes were reported in [26] and [27] respectively. Each 25 µL PCR mixture contained nuclease-free water, 1 X final concentration NH_4_ reaction buffer (Bioline), 0.2 mM final concentration deoxynucleoside triphosphate mix (ThermoFischer Scientific), 3 mM final concentration MgCl_2_ solution (Bioline), 0.3 µM final concentrations of each primer (forward and reverse) (ThermoFischer Scientific), 1 U of BioTaq DNA polymerase (Bioline) and 1 µL of the target DNA. In the absence of positive controls for *emrC* and *bcrABC* genes, PCR amplicons that aligned with the expected product sizes for these genes (in a previous study using this PCR primers and conditions) was re-sent for Sanger sequencing (Inqaba Biotechnical Industries) to confirm gene presence. Sequencing files were uploaded to National Centre for Biotechnology Information. Basic Local Alignment Search Tool (BLAST) analyses confirmed gene presence and this DNA was used as positive controls. The results aligned with subsequent WGS outputs (section 2.9). Molecular Biology Grade Water (HyClone™) was used as a negative PCR control, and a negative extraction control was included. PCR amplification was conducted using the T100 Thermal Cycler (Bio-Rad). The PCR cycling conditions for the *emrC* gene were according to [26]. The PCR cycling conditions for the *bcrABC* gene were according to [29]. Amplified PCR products were separated by electrophoresis using 1.5% agarose gel (Lonza) in 1 X TAE buffer supplemented with smart glow pre-stain (0.05 µL/mL) (Accuris). A 100 bp DNA ladder (ThermoFischer Scientific) was incorporated for the *emrC* gene and a 1000 bp DNA ladder (ThermoFischer Scientific) was used for the *bcrABC* gene cassette. Electrophoresis was conducted at 90 Volt for 90–120 min and images were captured using a Gel Doc™ XR+ instrument (Bio-Rad) with Image Lab™ Software.

### 2.8. Statistical analysis

Cross tabulation with the Fisher exact test was used to compare prevalence of attributes (e.g., lineage, antibiotic susceptibility, *bcrABC* gene) between clinical and seafood/FPE samples. Confidence intervals for proportions were calculated using the Binomial distribution. A single-sample test was conducted to assess the prevalence of attributes at a 50% occurrence rate using the binomial distribution as the underlying statistical model. Analyses were done using Statistica 14.0 and the R function “binom.test” in R version 4.3.1.

### 2.9. Whole genome sequencing

A subset of twenty-four isolates were processed by WGS based on the isolates' lineage distribution, factory origin and type of seafood product. Seven lineage I isolates and nine lineage II isolates were chosen from six different factory origins. The phenotypic antibiotic resistance results and the various isolation dates within factories were considered to ensure isolates from a range of years and resistance profiles were included in the study. All clinical isolates (n = 8) were chosen to be processed by WGS.

The same DNA extracted from *L. monocytogenes* (section 2.3) was used for processing by WGS. DNA quantity and purity (for WGS) were assessed using a NanoDrop™ 1000 spectrophotometer (Thermo Fischer Scientific, Waltham, MA, USA). The ratio of absorbance at 260 nm and 280 nm were determined, and a reading between 1.8 and 2.0 were accepted for DNA purity.

WGS was performed by CosmosID (Maryland, USA) (n = 20) and Inqaba Biotechnical Industries (Pty) Ltd. (n = 4). Detailed methods are noted below. FASTA (File for representing nucleotide (DNA and RNA) sequence data) and FASTQ (raw) files were generated from WGS and the FASTA files were uploaded to the Centre for Genomic Epidemiology (CGE) (bioinformatics tools and resources) and Institut Pasteur for analyses.

#### 2.9.1. Cosmos ID

DNA samples were quantified using the GloMax Plate Reader System (Promega) using the QuantiFluor® dsDNA System (Promega) chemistry. DNA libraries (on 20 isolates) were prepared using the Nextera XT DNA Library Preparation Kit (Illumina) and IDT Unique Dual Indexes with total DNA input of 1ng. Genomic DNA was fragmented using a proportional amount of Illumina Nextera XT fragmentation enzyme. Unique dual indexes were added to each sample followed by 12 cycles of PCR to construct libraries. DNA libraries were purified using AMpure magnetic Beads (Beckman Coulter) and eluted in QIAGEN EB buffer. DNA libraries were quantified using Qubit 4 fluorometer and Qubit dsDNA HS Assay Kit. Libraries were then sequenced on Illumina NovaSeq platform 2 x 150 bp. Raw paired end reads were trimmed and processed using BBDuk with read quality trimming parameter of 22. SPAdes was used to assemble the trimmed FASTQ files using the careful parameter. The completeness of the assembled isolate was evaluated using CheckM lineage_wf function.

#### 2.9.2. Inqaba Biotec

The DNA library (on 4 isolates) was prepared according to PacBio's Procedure-checklist-Preparing-whole-genome-and-metagenome-libraries-using-SMRTbell-prep-kit-3.0. High molecular weight genomic DNA was sheared to approximately 10kb fragments using Covaris g-Tubes, purified with AMPure® PB Beads, and the sheared DNA quantified using Qubit™. SMRTbell libraries were performed using PacBio's Microbial Multiplexing workflow. The resulting libraries were size selected using the BluePippin size selection system (10–18kb), quality control followed using Qubit™ and size distribution was analyzed using the TapeStation® system. Libraries were then prepared for sequencing following the online SMRTlink guided protocol. The samples were sequenced on the PacBio Sequel IIe system and assembled using the SMRTLink 10.1 software. The provider uses an in-house python script to convert files from FASTQ to FASTA format. We chose to process four samples separately with this local service provider since they were added after the previous batch had already been processed abroad (section 2.9.1). This service provider ensured a quicker turnaround time for these fewer samples.

#### 2.9.3. Serotype, sequence type (ST), and clonal complex (CC) assignment

The Multilocus sequence typing (MLST) profiles were determined by uploading the FASTA files to the CGE's MLST 2.0 tool for *L. monocytogenes* (https://cge.food.dtu.dk/services/MLST/) [58]–[64]. Serotypes were then inferred from the sequence type. Clonal complexes were determined by uploading FASTA files unto the BIGSdb-*Lm* database maintained at the Institut Pasteur (https://bigsdb.pasteur.fr/listeria/) [65].

#### 2.9.4. Identification of virulence genes

Virulence genes were identified by uploading the FASTA files to the CGE's Virulence finder 2.0 tool (https://cge.food.dtu.dk/services/VirulenceFinder/) for *Listeria* spp. The minimum percentage of nucleotides that are identical between the best matching virulence gene in the database and the corresponding sequence in the genome (threshold for % identity) was set to 90%. The number of nucleotides a sequence must overlap a virulence gene to count as a hit for that gene (minimum length) was set to 60% [64],[66],[67]. Additional virulence genes (LIPI-3 and LIPI-4) were screened for by uploading FASTA files to the BIGSdb-*Lm* database maintained at the Insitut Pasteur (https://bigsdb.pasteur.fr/listeria/) [65].

#### 2.9.5. Identification of plasmids

The FASTA files were uploaded to the CGE's Plasmid Finder 2.1 tool (https://cge.food.dtu.dk/services/PlasmidFinder/) for Gram-positive bacteria. The minimum percentage nucleotides that are identical between the best matching plasmid in the database and the corresponding sequence in the genome (threshold for minimum % identity) was set to 95% and the minimum percentage of coverage between a plasmid and uploaded data (minimum % coverage) was set to 60% [64],[68].

#### 2.9.6. Identification of resistance genes and other survival determinants

FASTA files were uploaded to CGE's ResFinder 4.1 tool (https://cge.food.dtu.dk/services/ResFinder/) for acquired antimicrobial resistance genes. The minimum percentage of identity between the best matching resistance gene in the database and the corresponding sequence in the genome (threshold for % identity) was set to 90%. The minimum length of coverage that a sequence must overlap a resistance gene to count as a hit for that gene (minimum length) was set to 60% [64],[69],[70]. To determine the presence of antibiotic resistant, sanitizer tolerant, and SSI genes, FASTA files were also uploaded to the BIGSdb-*Lm* database maintained at the Insitut Pasteur (https://bigsdb.pasteur.fr/listeria/) [65].

## 3. Results and discussion

### 3.1. Testing for hly gene presence using PCR

The *hly* gene was present in all forty-six *L. monocytogenes* isolates from seafood, FPE and clinical origin. The presence of this gene serves as confirmation of *L. monocytogenes* [71]. According to food regulation laws, *L. monocytogenes* must not be detected at time of production in products that can support the growth of this pathogen (e.g., RTE fish products). Furthermore, products containing stabilizing agents against the growth of *L. monocytogenes* or that cannot support the growth of *L. monocytogenes* during the shelf life are suggested to have less than 100 cfu/g (colony forming units per gram) throughout the shelf life of the product until the point of consumption [72].

### 3.2. Lineage characterization and potential contamination sources

Screening with enzymes *Nde*I and *Bfo*I classified all isolates into either lineage I or II. Of all the isolates (n = 46), lineage II accounted for 67% (n = 31) and lineage I accounted for 33% (n = 15) (Table S1). Isolates from clinical origin (n = 8) were all classified as lineage I. Lineages I and II were present in the seafood products (n = 32) and the FPE (n = 6) isolates. The isolates from seafood had a distribution of lineage I (n = 6) and lineage II (n = 26). The FPE isolates had a distribution of lineage I (n = 1; 17%) and lineage II (n = 5; 83%).

Lineage II was the most common genetic lineage observed in isolates from seafood and the FPE (n = 31; 82%) with a 95% confidence interval (CI: 66%–92%, Fisher exact p < 0.01). This high prevalence of lineage II in seafood and the FPE is consistent with other studies [5],[19],[21],[23],[38],[73],[74]. Other researchers, have however found lineage I to be more prevalent in fish products and the fish FPE [18],[75],[76]. A study by authors [18] in the South African setting found that lineage I was significantly more associated with the raw hake samples (95%) than lineage II. However, importantly, these findings were from one factory over many sampling occasions (which could allude to a persistent strain). In contrast, this current study included various seafood products and factory origins over the Western Cape, providing a broader overview of the lineage distribution in this specific industry.

Lineage I was the only lineage detected from the clinical isolates in this study (95%, CI: 69%–100%, p < 0.01). Lineage I has been associated with listeriosis cases for many years, and is reported in studies [18],[77]. However, recently, there is more evidence of lineage II isolates causing listeriosis around the world [5],[7],[78]. Fagerlund *et al*. [79] examined Norwegian clinical isolates, and found that 80% of the clinical isolates examined belonged to lineage II [79].

Four factory origins (A, D, E and F) consisted of isolates of one lineage type i.e., lineage II (Table S1). Factory B, C and G contained isolates of lineage I and II categories, indicating that different serotypes (across lineages) are contaminating these facilities.

The *L. monocytogenes* contamination on these products can come from a variety of sources and by understanding these contamination routes, future contamination can be prevented, and risk mitigation practices can be implemented. One possible route of contamination could be from the raw incoming fish, which is generally quite low, and varies from 0–10% [4],[76],[80]. If the raw incoming fish is contaminated it could result in contamination of the processing equipment and surfaces, which can be the beginning of a persistent problem in the FPE [76].

Furthermore, contamination from the FPE and equipment during and after processing of the final product have been found to be the main sources of contamination [4],[80]–[84]. Conveyor belts, gutting machines, trimming boards, weighing tables, smokehouses, packaging material, and personnel are all possible contributors to product contamination [74],[80]–[82],[84],[85]. The presence of *L. monocytogenes* in the FPE can indicate inadequate cleaning and sanitation processes or staff being poorly trained to implement these practises [4],[24],[86]. Cleaning and sanitation could be difficult due to harbourage sites like cracks, equipment design, and overall difficult to reach areas. However, these zones favor proliferation and survival of *L. monocytogenes* in the FPE and results in subsequent cross-contamination.

The cold-smoking process poses several challenges to the industry as limited heat is applied to the product and although a microbial reduction takes place, it is insufficient for complete elimination of *L. monocytogenes* [32],[87],[88]. Therefore, careful attention must be given to the cleaning and sanitation processes before proceeding with production to prevent any cross-contamination to a minimally processed product intended to be a RTE product.

### 3.3. Phenotypic resistance to antibiotics

All isolates were tested for susceptibility to seven antibiotics, with 98% displaying susceptibility to all the antibiotics tested (95%, CI: 95%–99%, p < 0.01). Further, all isolates (n = 46) were susceptible to ampicillin, gentamycin and meropenem (Table S1). All isolates from the clinical category (n = 8) were susceptible to six of the seven (6/7) antibiotics tested (95%, CI: 85%–99%) (p < 0.01): tetracycline, erythromycin, chloramphenicol, ampicillin, gentamycin and meropenem. Three (n = 3) of the clinical isolates were resistant to sulphamethoxazole/trimethoprim. No significant difference in susceptibility was found between clinical and seafood and FPE isolates (Fisher exact: p = 0.19).

There were five isolates from seafood and the FPE resistant to antibiotics tested: tetracycline (n = 2), sulphamethoxazole/trimethoprim (n = 2), erythromycin (n = 1), and chloramphenicol (n = 1). One isolate (Factory C) isolated from smoked trout roulades and categorised into lineage I was resistant to two antibiotics; tetracycline and sulphamethoxazole/trimethoprim (Table S1). Other studies also found resistance in *L. monocytogenes* isolates from fish and the fish FPE to these antibiotics with high percentages of resistance to tetracycline (27%–63%), sulphamethoxazole/trimethoprim (8.4%–44%), erythromycin (7.4%–87%), and chloramphenicol (23%) [18],[20],[84],[89]. Erythromycin is a treatment option for pregnant women with listeriosis [18],[23]. Sulphamethoxazole/trimethoprim is a second choice of treatment for listeriosis, especially for pregnant women [21].

### 3.4. *emrC* and *bcrABC* genes detected by PCR

*L. monocytogenes* is increasingly showing tolerance to QACs, benzalkonium chloride (BC), and other QACs commonly used in the FPE to keep it and other microorganisms in control [24]–[26]. QACs are the most common disinfectant used in the FPE, in households and as antiseptics in the clinical environment [28],[90]. *emrC* and *bcrABC* genes are associated with an increased BC tolerance and an increase in the minimum inhibitory concentration of BCs to *L. monocytogenes* [24],[26],[31],[32],[91]. Furthermore, *L. monocytogenes* has shown ability to tolerate sublethal concentrations of these disinfectants [29],[32], making the eradication of theses isolates more difficult.

**Figure 1.**
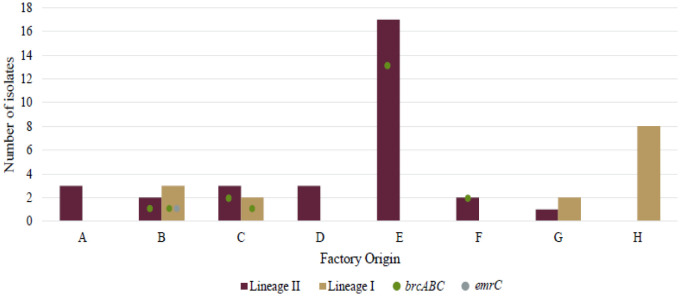
Lineage occurrence and presence of the *bcrABC* and *emrC* gene in *L. monocytogenes* from seafood products and the FPE (A–G), and clinical isolates (H). The coloured bars represent lineage I or II and their corresponding numbers on the y-axis. The circles/dots represent the genes (*bcrABC* or *emrC*) and their corresponding numbers on the y-axis. No dot signifies absence of these genes.

Twenty (43%) of the *L. monocytogenes* isolates (seafood, FPE and clinical) tested positive for the *bcrABC* gene cassette and one isolate tested positive for the *emrC* gene (Figure 1 and Table S1). Of the food and FPE isolates, 55% (n = 21) had a gene (*bcrABC* or *emrC*) present. None of the clinical isolates had either of the genes present. No significant difference was observed in the prevalence of the *bcrABC* gene cassette between the food and FPE categories (p = 87). However, genes *emrC* and *bcrABC* were significantly associated with seafood and the FPE, compared to the clinical isolates (Fisher exact; p < 0.01). Additionally, the presence of the *bcrABC* gene cassette was significantly more associated with lineage II isolates (p < 0.01). Many studies have found the presence of the *bcrABC and emrC* genes in *L. monocytogenes* isolates over a broad spectrum of food categories and FPEs [24],[29],[31],[32],[73],[74],[92].

The *bcrABC* gene was found in isolates from factories B, C, E and F (Figure 1). Factory E processes and manufactures cold-smoked salmon, salmon, trout, and tuna (slices, strips or fillets) (Table S1). From this factory, it was observed that over July to November 2021, various seafood products tested positive for *L. monocytogenes* lineage II. This can indicate *L. monocytogenes* spread through the factory or one specific site where the products are contaminated repeatedly. Furthermore 76% (13/17) of these isolates tested positive for the *bcrABC* gene cassette, which can contribute to a higher tolerance to BC used in the factory. It could be the same strain persisting, but confirmation would require SNP or cgMLST analysis. Where tolerance is detected, a practical solution could be to alternate a QAC based disinfectant with another type of disinfectant using a different mechanism of action (peracetic acid or chlorine) once to twice a week [29]. Poor factory design could also assist with tolerance to sanitizers. Recent research has reported that it is not one single gene or gene cassette influencing tolerance to disinfection or favoring persistence within a FPE, but rather the combination of genetic and non-genetic factors [31],[91].

Factory B and C contained both lineage I and II isolates with the *bcrABC* gene, indicating gene presence in different strains across lineages. A lineage I fish isolate from Factory B contained the *emrC* gene (Figure 1). The *emrC* gene encodes a QAC efflux protein pumping QACs out of the cell and allows for increased BC tolerance, increased capacity to form biofilms, increased virulence capacity, and reduced susceptibility to antibiotics (e.g., amoxicillin and gentamycin) [26],[30]. The *emrC* gene was originally present and isolated from ST6 isolates [26] and has been isolated from many sequence types and lineages ever since [73],[74].

Researchers [93] found the presence of *emrC* (4.2%) and the *bcrABC* gene cassette (2.1%) in fish and fish FPE isolates from Poland (n = 287). Additionally, in a study [92], the *bcrABC* gene cassette (19%) and the *emrC* gene (15%) were identified in samples from three different FPEs (n = 100), finding that most *bcrABC* was associated with lineage I isolates and *emrC* with lineage II isolates. The presence of the *bcrABC* gene cassette was related to a multi-state outbreak in the USA (1998–1999) associated with hot dogs [27],[94]. In relation to these studies, we observed a 43% prevalence of the *bcrABC* gene cassette in a smaller subset (n = 46) of food, FPE, and clinical isolates (with 53% associated with food and the FPE).

The presence of these genes (*emrC* and *bcrABC*) in isolates from seafoods and the FPE (with known lineage data) may provide insights to food safety managers as to why isolates persist within the FPE. These genes may confer adaptive ability to isolates in harsh environmental conditions where they are known to persist. This may also explain why isolates from clinical origin are less likely to contain stress tolerance genes (with a lower prevalence in food factories). It shows the need for more surveillance and the importance for implementing effective mitigation processes for risk management [91].

### 3.5. Whole genome sequencing - serotype, sequence type, and clonal complex assignment

Of the twenty-four isolates subjected to WGS, three serotypes, ten STs, and eight different CCs were identified (Table 2). Among the 16 *L. monocytogenes* isolates from seafood and the FPE, eight STs from three serotypes were identified: Serotype 1/2a (56%), 1/2b (25%) and 4b (19%). The results are in agreement with global research on seafood, with serotype 1/2a being the predominant serotype followed by serotypes 4b, 1/2b and 1/2c [7],[19],[20],[23],[87],[95]. From the 13 described serotypes of *L. monocytogenes*, the majority of listeriosis cases are caused by serotypes 1/2a, 1/2b and 4b with the latter being associated with outbreaks and responsible for 50–60% of clinical cases [9],[11],[14],[43],[96].

**Figure 2.**
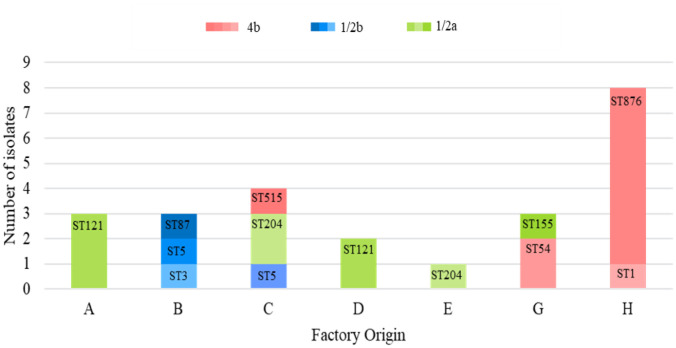
Distribution of sequence type (ST) across the factory origin of seafood and the FPE (A, B, C, D, E, G) and clinical (H).

Lineage II, serotype 1/2a (Figure 2) comprised of three STs: ST121 (n = 5) (Factories A and D), ST155 (n = 1) (Factory G) and ST204 (n = 3) (Factory C and E). Lineage I, serotype 1/2b, included three STs: ST3 (n = 1) (Factory B), ST5 (n = 2) (Factory B and C) and ST87 (n = 1) (Factory B), whereas serotype 4b included two STs: ST515 (n = 1) (Factory C) and ST54 (n = 2) (Factory G).

#### 3.5.1. Factory A and C

Factory A comprised of serotype 1/2a, ST121, CC121 isolated on three different occasions over the years 2019 and 2021 from cold smoked trout ribbons and salmon ribbons (Table 2). CC121 (Factories A and D in this study) has been the most common CC identified in many fish factories [5],[38],[73],[77] and has shown persistence in many FPEs in many European countries [77],[97]–[99]. *L. monocytogenes* CC121 has often been isolated from immunocompromised patients in France [98] and has also been found to be the second most common CC among Norwegian clinical isolates [5],[74]. For all the seafood/fish included in this study, the distribution site is known, but the origin of the fish itself is unspecified.

Factory C had contamination with all 3 serotypes; serotype 1/2a (ST204, CC204, n = 2) (fish room floor and smoked trout terrine), serotype 4b (ST515, CC1, n = 1) (smoked trout roulades) and serotype 1/2b (ST5, CC5, n = 1) (smoking room drain). ST204 was isolated on two different occasions over years 2019 and 2021 (Table 2). ST121 and ST204 are regarded as two of the most common STs found in the FPE [22],[100]. ST204 is one of the most prevalent STs isolated in Australia, found in a diverse range of foods, environments and has also been associated with human clinical infections [100]. The ST515 isolate from smoked trout roulades is grouped into CC1, a hypervirulent complex of *L. monocytogenes*. CC1 was reported as one of the most common CCs in a salmon processing facility in Norway and was also persistent in these factories, showing it does have the ability to persist and survive in seafood and the FPE [5],[74]. This CC1 holds clinical significance, causing disease and being associated with clinical cases globally [5],[73],[77],[98]. In a study comparing *L. monocytogenes* isolates from food, FPE and clinical cases in the European Union, CC1 was the most prevalent from clinical cases [77].

It was speculated that there might be isolates from Factory A to Factory G that could possibly be the same ST (strain) within the respective factories. Studying the WGS data revealed that Factory A and C (Table 2) had the same STs detected in samples collected over multiple years. Sequence types from Factory A (ST121), and C (ST204) were isolated multiple times over a three-year period (2019 and 2021) suggesting a persistent strain. However, confirmation would require SNP analysis or cgMLST analysis. A persistent strain is defined as a specific subtype of *L. monocytogenes* isolated repeatedly from the same environment over an extended period of time (6 months or longer) and several cases of persistent strains have been reported in many seafood processing facilities [4],[80],[82],[86],[87]. ST121 has been shown in many studies to persist in the FPE for months or even years [40],[77],[97]–[99],[101]. Persistent strains can be present due to several factors, including difficult to clean areas, the introduction of a resistant strain, the adaption of a strain to the selection pressures in the FPE, and conditions that promote the growth and survival of these strains [86],[87]. It can also be the result of poor cleaning and sanitation protocols or inadequate implementation of these protocols [4],[24],[86]. A persistent strain may also be due to the same strain entering the FPE from the raw materials being reintroduced repeatedly between sampling events [74] i.e., repeated introduction from an outside reservoir.

#### 3.5.2. Factory B

Three isolates from this factory were identified as lineage I (Table S1). WGS revealed that it was three different STs/strains (Table 2). These three isolates (fish types unknown) belonged to serotype 1/2b: ST87(CC87), ST3(CC3), and ST5(CC5). With three different STs isolated from fish within three months suggests that the contamination is from incoming raw materials or packaging material. Furthermore, the ST5 isolate from the smoking room drain was not detected in the fish isolates but only from one environmental isolate. This could be explained by the small sampling set but may allude to the possibility of other fish products being contaminated with ST5 too.

CC87 was one of the predominant CCs isolated in fish foods in China [102] and has been isolated in fish and fish FPE in European countries [77]. This CC has been reported as one of the most prevalent CCs from clinical samples in China [102]. Moreover, ST87 strains from clinical origin in China have been reported to be involved in maternal-neonatal infections (n = 6) and a central nervous system infection (n = 1) [103].

CC3(ST3) (fish) (Table 2) is one of the oldest, most prevalent, epidemic clones [104]. CC3 has been ranked among the 4 most common clones (CC1, CC2, CC3 and CC9) over five continents [105], the fourth most common CC in Europe and is predominant in Australia. It has also been found in South and North America, Japan and Oceania and identified as a prevalent CC in the Republic of Serbia [105]–[107]. ST3 was also reported as the second most common ST isolated from food of animal origin in Poland from 2013–2016 [108]. Additionally, in the United States, CC3 was responsible for a large outbreak of febrile gastroenteritis because of contaminated chocolate milk in 1997 [109],[110]. ST3 has also been found to persist in two king oyster mushroom production plants [111]. CC3 and CC5 have been found in many seafood processing facilities and seafood products [31],[73],[77],[91],[102].

Researchers [91] reported CC5 to be the most common CC among 1279 *L. monocytogenes* isolates from various foods and FPEs in Canada. In Beijing, China, ST5 was one of the most common STs isolated from clinical listeriosis cases [112]. Majority of these ST5 strains were isolated from patients with pregnancy associated infections and all of the foetuses died [112]. Furthermore, CC5(ST5) have been implicated in several outbreaks related to cantaloupe, ice cream and stone fruit in the US [112],[113]. Researchers [114] reported that CC5 and CC121 (recovered in Factories A and D in this study) are among the CCs that are increasing in frequency, reported globally in listeriosis cases.

#### 3.5.3. Factory D, E and G

ST121 (n = 2) was the only serotype identified in Factory D from salmon and oysters on the same day. This suggests cross-contamination as these two products differ, with different processing methods. The same ST may also indicate that the products were contaminated at the same point. The packaging area, materials, or food production staff may have been a possible source of this mutual contamination.

Factory E's one isolate belonged to ST204. Not enough samples for Factory E were processed by WGS for further discussion. Factory G had one serotype 1/2a (ST155, CC155) and two serotype 4b (ST54, CC54) isolates all from fresh hake on the same day (Table 2 and Figure 2). CC155 isolates were found in low numbers in fish foods in China, as well as being one of the most prevalent CCs from various FPEs in Canada and the United States [31],[91],[102]. CC155 was also isolated from a fish manufacturer in Poland [38] and in FPEs in Europe [77].

#### 3.5.4. Clinical isolates and clonal complex 1

Analysis of the 8 clinical isolates collected in 2019 from patients with listeriosis revealed that all the isolates belonged to serotype 4b, CC1 consisting of two STs (ST876 and ST1) (Figure 2 and Table 2). The predominant sequence type isolated was ST876 that was found in 87.5% (n = 7) of the samples. One ST1 isolate was identified. ST1 has been identified as a dominant subgroup at global level with strong clinical significance [100]. CC1 has been reported to be the most prevalent CC isolated from clinical cases in the European Union [77] as well as in Norwegian clinical cases [5]. CC1 is a hypervirulent complex of *L. monocytogenes*, meaning clones of *L. monocytogenes* most likely to cause disease especially to the central nervous system or maternal-neonatal listeriosis [73],[98]. Furthermore, CC1 is able to colonise the intestinal lumen better and invade more intestinal tissue than hypovirulent strains [73]. Other hypervirulent complexes of *L. monocytogenes* identified in other studies include: CC2, CC4 and CC6 [73],[98].

ST1 and ST876 were not isolated from any seafood or FPE isolates from this study. However, of the eight CCs identified in this study (seafood, FPE and clinical), all have been implicated in clinical cases around the world [74],[79],[95],[102],[106],[115]–[117]. Eight of the ten STs identified in this study (ST1, ST3, ST5, ST87, ST121, ST155, ST204, ST876) were found in another South African setting done on the red meat and the poultry value chain [22],[118]. Moreover, ST1, ST3, ST5, ST54, ST87, ST155, ST204, and ST876 (eight of the ten STs from this study) were also among the STs associated with clinical cases during the South African listeriosis outbreak in 2017 to 2018 [115].

### 3.6. Identification of virulence genes and *Listeria* pathogenicity islands

The pathogenicity and virulence of *L. monocytogenes* is determined by the presence of a large number of virulence genes, with varying functions at various stages of its life cycle infecting humans or animals [119]. A total of 91 different genes were identified within the twenty-four sequenced isolates using the CGE's Virulence finder 2.0 tool. Seventy-eight of these genes were present in all isolates (core virulence genes) regardless of the lineage group, serotype, ST or CC: *actA*, *agrA, flaA, flgC, flgE, gadB, gadC, lap, lapB, oatA, oppA, orfX, orfZ, rli55, rli60, rsbv, biLE, bsh, btlB, chiA, clpB, clpc, clpe, clpp, codY, ctaP, ctsR, dal, degU, dltA, fbpA, fri, fur, hfq, hly, htrA, hupC, iap, inlA, inlB, inlC, inlH, inlJ, lgt, lhrC, lipA, lmo0514, lmo2085, lntA, lpeA, lplA1, lsp, mogR, mpl, mprf, murA, perR, pgdA, pgl, plcA, plcB, prfA, prsA2, pycA, recA, relA, secA2, sigB, sipX, sipZ, sod, srtA, srtB, stp, svpA, tcsA, tig*, and *uHpt*.

All 24 isolates contained the LIPI-1 (*actA, hly, mpl, plcA, plcB*, and *prfA*) and LIPI-2 (*inlA, inlB, inlC and inlJ*) (Table 2) genes. Other researchers found the same [39],[43],[120],[121]. However, others reported absence of some of the genes within LIPI-1 and LIPI-2 [99],[118]. These genes play key roles in the pathogenic and virulence processes of *L. monocytogenes:* cell invasion process, proliferation and intra- and intercellular bacterial movement.

#### 3.6.1. *Listeria* pathogenicity island 3

**Figure 3.**
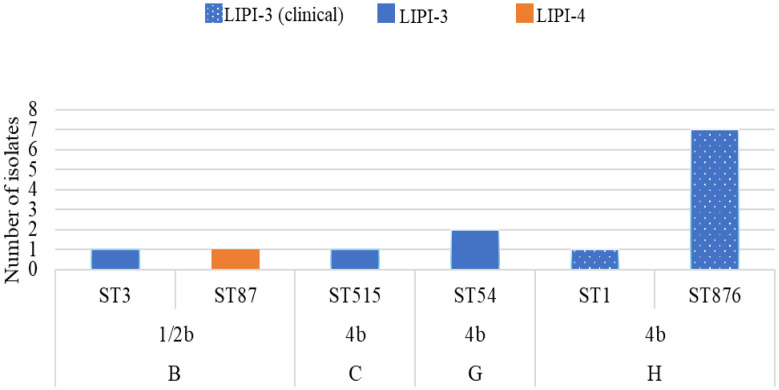
The LIPI-3 and LIPI-4 occurrence over serotype and sequence type from seafood and the FPE (B, C, G) and clinical origin (H).

**Figure 4.**
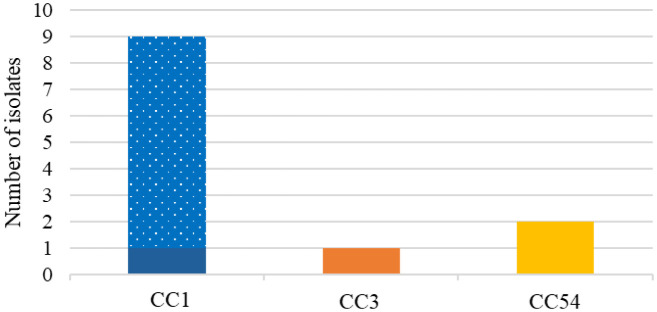
The LIPI-3 occurrence over clonal complexes (CC) from seafood, the FPE and clinical origin (B, C, G and H).

LIPI-3 was found in twelve (n = 12, 50%) isolates (Figure 3 and Table 2) from only lineage I (ST1, ST3, ST54, ST515 and ST876). From the eight LIPI-3 genes (*llsA; llsB; llsD; llsG; llsH; llsP; llsX; llsY*), five (*llsA, llsD, llsH, llsP, llsY*) were present in all the twelve isolates. Nine of 12 isolates had the full LIPI-3. *llsB* was absent from a ST3 fish isolate, *llsG* was absent from a ST876 clinical isolate and *llsX* was absent from a ST1 clinical isolate. The absence of the *llsX* gene in this ST1 isolate from clinical origin is quite interesting as many studies have used the presence of *llsX* as a marker for LIPI-3 presence, and to infer the production of listeriolysin S [34],[103],[122]. Painset *et al*., [77] also noted that some isolates lack specific LIPI-3 genes but have an otherwise intact LIPI-3 structure. In our study, eight (n = 8, 66.6%) of the isolates containing LIPI-3 were from clinical origin. The four remaining isolates originated from seafood products: fish (ST3), fresh hake (ST54-2 strains), and RTE smoked trout roulades (ST515) (Table 2 and Figure 3). LIPI-3 was only present in CCs from lineage I (CC1, CC3, CC54) (Figure 4). Previous studies have reported LIPI-3 from the same CCs [33],[35],[38],[77],[120],[122]. Many researchers have found that LIPI-3 are more prevalent in clinical and or lineage I strains and absent from lineage II [5],[33],[38],[77],[123]. This could explain why lineage I is overrepresented in the clinical cases worldwide. This also highlights the risk posed to consumers by seafood products in this study that contain LIPI-3.

#### 3.6.2. *Listeria* pathogenicity island 4

LIPI-4, containing six genes (LM9005581_70009, LM9005581_70010, LM9005581_70011, LM9005581_70012, LM9005581_70013, LM9005581_70014), encodes for a cellobiose-family phosphotransferase system found in hypervirulent CCs. LIPI-4 was only present in one isolate, lineage I (ST87), found in fish from Factory B (Figure 3 and Table 2). Painset *et al*. [77] found that 0.07% (n = 81) of their data set (n = 1143) contained the LIPI-4. All the isolates from their study harbouring LIPI-4 were from ST87 and ST4, from clinical and food origin. LIPI-4 was found in ST87 from food and clinical isolates in other regions [77],[118],[120] and has shown to be present almost exclusively in lineage I isolates [5]. Additionally, LIPI-4 has been isolated from CC87 (n = 6) strains implicated in listeriosis in China, causing maternal-neonatal listeriosis and infection in the central nervous system [103]. ST87 is a known hypervirulent strain in China [103],[122],[124]. This highlights the risk of the ST87 isolate from fish (Factory B) containing LIPI-4.

Two strains from Factory B (fish) contained LIPI-3 (ST3) and LIPI-4 (ST87) genes, respectively. Both these LIPIs contribute to hypervirulence, and the presence of these genetic factors is therefore of high clinical significance. The presence of these LIPIs in the fish isolates may suggest that there are more isolates containing these genes in Factory B, which highlights the concern and risk for cross-contamination.

### 3.7. Identification of plasmids

Plasmid incompatibility groups were identified in 81% (n = 13) of the seafood and FPE isolates (Table 2). No plasmids were present in the clinical isolates or fresh hake from Factory G (ST54 and ST155). Three different plasmid groups were identified: Inc18 (rep25), Inc18 (rep26), and Rep_trans (repUS43). Plasmid group Inc18 (rep25) and Inc18 (rep26) have been identified in *L. monocytogenes* by others [73],[74],[125]. All the plasmids identified were part of the repA-family, theta-replicating plasmids [74],[126]. The rep_trans (repUS43) is a rolling-circle replicating type plasmids and has been previously isolated from *Enterococcus faecium* [127],[128]. Eleven isolates carried the plasmid incompatibility group Inc18(rep26) from lineages I and II (ST121, ST204, ST5, ST87). One lineage I isolate (ST3) carried the plasmid group Inc18(rep25) whilst one isolate (ST515) carried the plasmid group Rep_trans (repUS43).

Five different plasmid types were identified among them: plasmids pLM5578 (n = 5), pLGUG1 (n = 1), N1011A (n = 5), pLM33 (n = 1) and DOp1 (n = 1). All ST121 isolates (n = 5) contained the plasmid pLM5578. Several researchers have found this plasmid in many ST121 isolates [119],[126],[129],[130]. The plasmid N1011A was found in all ST204 (n = 3) and ST5 (n = 2) isolates. This plasmid has been found in cooked shrimp from Chile (ST5) and in ST204 from a FPE in South Africa [119],[125]. Furthermore, in the present study, plasmid pLGUG1 was found in ST87 from fish, plasmid pLM33 in ST3 and plasmid DOp1 in the ST515 isolate from RTE smoked trout roulades (Table 2).

Plasmids, pLM5578, pLGUG1, N1011A, and pLM33 have all been found in *L. monocytogenes* isolates with varying STs [119],[125],[126],[129]–[131]. Plasmids pLM5578 and pLM33 have shown the presence of cadmium-transporting ATPase (cadA) and of a cadmium efflux system accessory protein (cadC) associated with cadmium and heavy metal resistance in other studies [131],[132]. Furthermore, it has been previously noted that resistance to cadmium is also associated with tolerance to QACs contributing to *L. monocytogenes'* persistence in FPEs [132]. Genes found on plasmid pLGUG1, encode for a MATE (multidrug and toxic compound extrusion) family multidrug efflux pump [130]. The presence of these plasmids contributes to an increased tolerance to multiple stresses encountered in the FPE aiding in the survival of these strains in the environment [119].

### 3.8. Identification of antibiotic resistance genes

Screening the WGS data for antimicrobial resistance genes revealed the presence of intrinsic resistance genes in all isolates: *fosX* (fosfomycin resistance thiol transferase), *lmo0919* (antibiotic ABC transporter ATP-binding protein, lincosamides), *mprF* (phosphatidylglycerol lysyltransferase; responsible for bacterial peptide resistance), *norB* (multidrug efflux pump; quinolones), and *sul* (dihydropteroate synthases; sulfonomides). These were also reported in other studies [35],[36],[38],[125].

**Figure 5.**
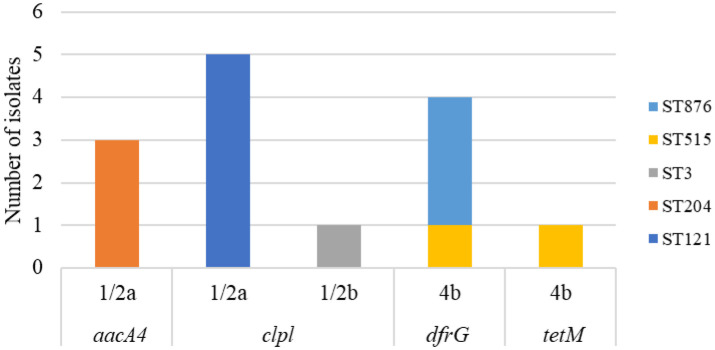
Detection of antibiotic resistant genes by WGS across serotype and sequence type of *L. monocytogenes* from seafood products, the FPE and clinical origin.

All ST204 isolates (n = 3; Factory C and E) (Figure 5) tested positive for the *aacA4* gene, responsible for resistance to aminoglycosides (like gentamycin). Aminoglycosides are often used in combination with ampicillin or penicillin as first choice of treatment for listeriosis [9],[22],[36]. However, none of these isolates showed phenotypic resistance to gentamycin (section 3.3; Table 2). The *aacA4* gene was found in various STs from the sediment of tilapia (farmed fish) ponds in Southern China [133] and in *L. monocytogenes* from fish and the fish FPE in Poland [38].

Genotypic and phenotypic antibiotic resistance mismatches have been recorded by others in *L. monocytogenes*, *Salmonella* and *Vibrio parahaemolyticus* [134]–[136]. Several factors can play into this mismatch [134]–[137]. Resistant genes that are plasmid encoded, may get lost during storage and sub-culturing, resulting in phenotypic susceptibility [134],[136]. Resistant genes found in WGS data but not correlating with phenotypic resistance might be silent/unexpressed genes [134],[135]. When these genes are not expressed they do not confer any selective advantage to the isolate [135],[137]. These unexpressed genes normally become transcriptionally active in rare cases or when the environment conditions allow [135],[136].

Phenotypic antibiotic resistance (section 3.3) was found in seven isolates, which were further processed by WGS (Table 2). Three of the clinical isolates (ST876) showed phenotypic resistance to sulphamethoxazole/trimethoprim. These three isolates all contained the *dfrG* gene (Figure 5) that is part of the folate pathway antagonist conferring resistance to trimethoprim.

ST515 showed phenotypic resistance to sulphamethoxazole/trimethoprim and tetracycline (Table 2). WGS data for this isolate showed *dfrG* and *tetM* genes (Figure 5), which confer resistance to these antibiotics. Gene *tetM* confers resistance to doxycycline, tetracycline and minocycline [64],[69],[70].

Furthermore, all ST121 isolates, and ST3 contained the *clpL* resistance gene, conferring increased tolerance to heat stress (Figure 5 and Table 2). This gene has been identified in *L. monocytogenes* in other studies [24],[32],[74],[126] and found in a range of CCs (CC3, CC5, CC7, CC9, CC31, CC121, CC199, and CC415) from clinical, environmental and food origin [74],[125],[138]. The ClpL protein (*clpL)* has been found in some *Listeria* plasmids before and acts as a stress response chaperone for the stress response regulator, CtsR [138]. Studies have found the *clpL* gene harboured on plasmids (e.g. pLM58 and pLM6179) may mediate resistance [126],[139]. A study [139] found that when this gene was introduced into a heat sensitive *L. monocytogenes* strain, it enabled survival at high temperatures. Additionally, other studies have hypothesised that ClpL proteins may aid survival in other stressors, like growth advantage in low pH or high NaCl [126]. This suggests why ST121 isolates are so frequently encountered and reported in food production environments worldwide. The *clpL* gene, SSI-1 and SSI-2 are found significantly more in isolates classified as pervaders (i.e., strains having an enhanced ability to spread and migrate to new locations or ecological habitats) [74].

### 3.9. Benzalkonium chloride and other stress tolerance genes

#### 3.9.1. Stress survival islands

**Figure 6.**
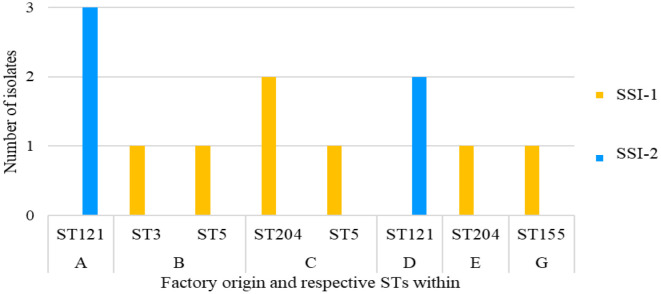
The occurrence of stress survival islets, SSI-1 and SSI-2 in sequence types from seafood products and FPEs across factory origins A–G.

Twelve (75%) isolates from seafood and the FPE contained SSI-1 or SSI-2 genes (Table 2). SSI-1 was found in 44% of isolates from seafood and the FPE (from factories B, C, E, G) (Figure 6). All five genes of the SSI-1 (*lmo0444, lmo0445, lmo0446 (pva), lmo0447 (gadD1), lmo0448 (gadT1*)) were observed in seven *L. monocytogenes* isolates; namely, ST155, ST204 (n = 3), ST3 and ST5 (n = 2) (Figure 6). It has been reported that SSI-1 is found in diverse STs and lineages, whereas SSI-2 is found mostly in lineage II, ST121 isolates [40],[43],[92], as observed in this study too. SSI-1 has been identified in ST3, ST5, ST155 and ST204 by other researchers [31],[43],[73],[77],[92]. The SSI-2 genes (*lin0464* and *lin0465*) were found in all (n = 5) ST121 isolates (Factory A and D) (Figure 6 and Table 2).

**Table 2.**
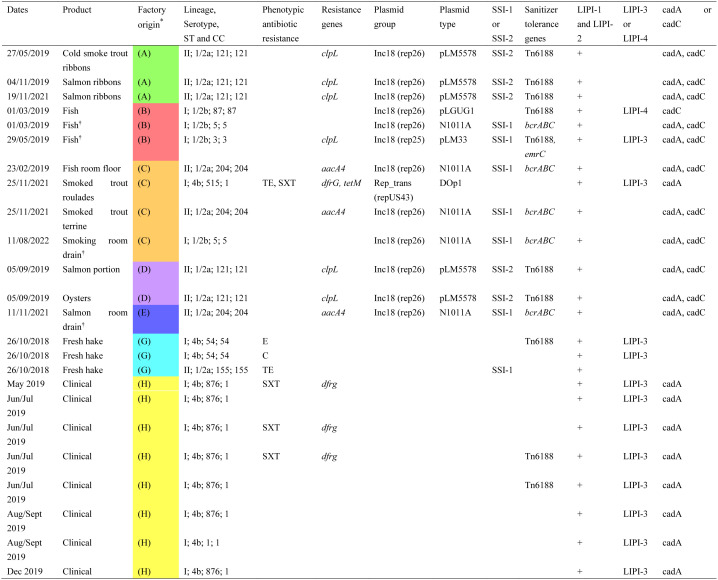
Phenotypic resistance and WGS profiles of isolates from seafood, the FPE and clinical origin.

* Colour coding identifies different factories recruited to the study. ^†^These four isolates underwent WGS by Inqaba Biotechnical Industries, the rest by Cosmos ID. The sequencing data underwent processing using two separate platforms from different service providers, resulting in assemblies that are not directly comparable. However, given the study's objectives and the focus on highlighted genes, this approach did not adversely affect the findings. **TE**: Tetracycline; **E**: Erythromycin; **C**: Chloramphenicol; **CN**: Gentamycin; **SXT**: Sulphamethoxazole/Trimethoprim; **MEM**: Meropenem. **LIPI-1** (*prfA, plcA, hly, mpl, actA, plcB*), **LIPI-2** (*inlABCJ*), **LIPI-3** (*llsAGHXBYDP*) and **LIPI-4** (*LM9005581_70009, LM9005581_70010, LM9005581_70011, LM9005581_70012, LM9005581_70013, LM9005581_70014*), **SSI-1** (*lmo0444, Lmo0445, lmo0446 (pva), lmo0447 (gadD1), lmo0448 (gadT1*)), **SSI-2** (*lin0464, lin0465(pfpl)*, **cadA** cadmium-transporting ATPase, and **cadC** cadmium efflux system accessory protein.

SSI-1 provides *L. monocytogenes* growth advantage in various environmental conditions like acidic, osmotic, gastric and bile stress and high salt concentrations encountered in food and the FPE [32,38–41,43]. SSI-2 helps *L. monocytogenes* grow better under alkaline and oxidative stress conditions [32],[39],[40]. Both SSIs aid in the survival and persistence of *L. monocytogenes* in FPEs when exposed to environmental pressures.

SSIs were found in all (n = 9) the lineage II isolates from this study. The SSIs were found in three of the seven lineage I isolates from seafood and the FPE (Figure 6 and Table 2) and absent in all the clinical isolates. All strains containing either LIPI-3 or LIPI-4 did not present with an SSI, except ST3 (Factory B) (Table 2). The presence of the SSIs could explain why lineage II isolates have a tropism to the FPE and are more prevalent in these environments than lineage I isolates. A study [36] reported no SSIs in the two strains from human listeriosis. However, they both harboured LIPI-3 and or LIPI-4.

#### 3.9.2. Benzalkonium chloride tolerance

Several genes (*qacH* (Tn6188)*, bcrABC, emrE*, *emrC, qacA, qacC*) have been identified in *L. monocytogenes* conferring tolerance to QACs like BC. These encode for QAC efflux pump systems that expel QACs from the intracellular environment to the outside of the cell preventing them to reaching their target [140],[141]. QACs are the most common disinfectant used in FPEs [28],[90].

**Figure 7.**
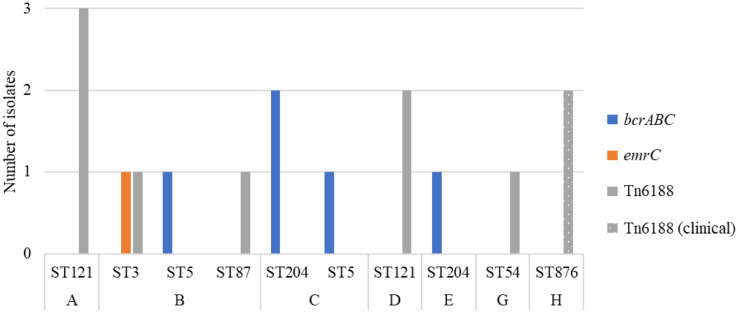
The occurrence of genes (*bcrABC*, Tn6188 *(qacH)* and *emrC*) conferring tolerance to QACs across sequence types from factory origins (A–G) (seafood and the FPE) and clinical (H).

Analysis of the WGS data (n = 24) towards the genetic factors responsible for tolerance to QACs, including BC (Table 2), revealed that 42% (n = 10) of isolates carried the transposon Tn6188_*qac (ermC)* and 21% (n = 5) of isolates carried the *bcrABC* gene cassette. The *emrC* gene was identified in one isolate from fish (ST3) (Figure 7 and Table 2). Sixty-three percent (n = 15) of the isolates harboured either the *bcrABC* cassette, the transposon Tn6188*_qac (ermC)* or *emrC*.

Transposable elements, or jumping genes, are a DNA sequence that has the ability to move from one location on the genome to another [142]. The movement of transposons from one microorganism to another is through horizontal gene transfer [142]. Transposon Tn6188 that encodes for QacH, is a transporter responsible for increased tolerance to BC [90],[143]. The transposon Tn6188*_qac (ermC*) was present in three serotypes over a range of STs (ST3, ST54, ST87, ST121, ST876) (Figure 7). Furthermore, 50% (n = 8) of the seafood and FPE isolates contained this transposon. Researchers [38] found that 43% of isolates from fish and the fish FPE in Poland contained this transposon. It has been found in ST121 isolates from seafood and various other FPEs [73],[86],[144]–[146], which can explain the widespread persistence of ST121. Additionally, other studies have identified *qacH* in other STs [24],[77],[146]. In this study, all the ST121 isolates carried transposon Tn6188 *(qacH)*, along with SSI-2 genes. Two clinical isolates (ST876) carried transposon Tn6188.

The *bcrABC* gene cassette was present in 31% (n = 5) of seafood products and the FPE (Table 2 and Figure 7) from (Factories B, C and E) in ST204 (n = 3) and ST5 (n = 2). This gene cassette was not present in any clinical isolates (origin H). The *bcrABC* gene cassette has been found in a range of STs and CCs including CC5 and CC204 [77],[91]. The *bcrABC* gene cassette has been found in low numbers from *L. monocytogenes* in RTE meat and the FPE in ST5 (2%; n = 1) [147], different foods in Switzerland (2%; n = 3) [146] and from fish and the fish FPE in Poland (2%; n = 7) [93]. Furthermore, the *bcrABC* gene was found in 35% of ST14 persistent strains in a rabbit meat processing plant [101] showing the contribution of this gene to the persistence and survival of *L. monocytogenes* in the FPE. This study found the gene cassette absent in ST121, mirroring findings from other studies [77],[91], though it has been detected in ST121 in some research.

The *emrC* gene was found in one isolate from fish categorised as ST3 from Factory B. This same ST3 strain also harboured the transposon Tn6188 (Figure 7). The *emrC* gene has been found in environmental ST5 isolates, CC7 isolates from listeriosis cases, and in serotype 1/2a isolates from fish products and the fish FPE [73],[79],[147]. The *emrC* gene has been associated with an increased incidence of ST6 listerial meningitis in the Netherlands [30],[148]. Additionally, the *emrC* gene was found in 5% (n = 21) of the listeriosis cases linked to meningitis with ST6, ST8, ST9, ST101 and ST576 [30]. The ST3 isolate in fish carried genes for SSI-1, LIPI-3, *emrC*, and transposon Tn6188 (Table 2), which enhance *L. monocytogenes*' survival and persistence in stressful environments. When present in a final product and consumed, it has an increased capability to invade the host (LIPI-1, 2, and 3) and cause listeriosis.

A study [74] reported the highest prevalence of SSIs and resistance genes in isolates from the FPE, consistent with the findings in this research. SSIs and genes promoting sanitizer tolerance help strains to survive in FPEs that seems inhospitable [149].

### 3.10. General trends across factories and clinical

*L. monocytogenes* from all six food factories (A–G) and clinical (H) presented with genes exhibiting sanitizer tolerance. All food factories (A–G) contained isolates carrying SSI-1 or SSI-2. LIPI-1 and LIPI-2 were present in all isolates across origins A-H. Lineage I isolates from three food factories and all clinical isolates contained LIPI-3 genes, and LIPI-4 genes were limited to one isolate/food factory.

These results highlight the mechanisms that enable *L. monocytogenes* to survive and persist in the FPE. The presence of LIPI-3 and LIPI-4 in seafood products and the FPE highlights the risk factors associated with these minimally processed RTE foods.

## 4. Conclusions

We are the first to characterize *L. monocytogenes* from seafoods and its food processing environment in South Africa (specifically the Western Cape) using WGS. It has provided valuable information on strain diversity, virulence potential and survival mechanisms of *L. monocytogenes*. The focus of the study was to shed light on the characterization of *L. monocytogenes* in seafood and address the existing research gaps within the South African context. Although the samples were exclusively collected from the Western Cape region, the study provided valuable insights that can serve as a foundation for future research opportunities and contribute to global knowledge.

We took a One Health approach looking at clinical, food and environmental domains to enhance the understanding of associated risks and improve intervention designs. The risk escalates because numerous seafood products are ready-to-eat, necessitating no further heating, while *L. monocytogenes* can persist and multiply at refrigeration temperatures. This is particularly concerning for immunocompromised individuals, and pregnant women. Further, the insights derived from this study can establish a fundamental understanding specific to our country. This understanding can serve as a benchmark for future assessments, enabling us to track the dissemination and evolution of these isolates along with their distinct genetic traits in the years to come. By gaining knowledge about the survival and persistence mechanisms of these isolates within the factory setting, food safety managers can make informed choices and institute efficient cleaning protocols to bolster food safety. This information thus significantly contributes to food safety decision making and risk assessment.

## Use of AI tools declaration

The authors declare they have not used Artificial Intelligence (AI) tools in the creation of this article.


