# Supplementary material for: Genetic diversity of Listeria monocytogenes from seafood products, its processing environment, and clinical origin in the Western Cape, South Africa using whole genome sequencing
Source: AIMS Microbiol. 2024 Aug 7;10(3):608–43. doi: 10.3934/microbiol.2024029 (PMC11362271; doi:10.3934/microbiol.2024029)
Supplement: Supplementary file 1 [file microbiol-10-03-029-s001.pdf]

AIMS Microbiology

AIMS Microbiology, 10(3): 608–643.
DOI: 10.3934/microbiol.2024029
Received: 25 April 2024
Revised: 08 July 2024
Accepted: 25 July 2024
Published: 07 August 2024

https://www.aimspress.com/journal/microbiology

Research article

Genetic diversity of *Listeria monocytogenes* from seafood products, its processing environment, and clinical origin in the Western Cape, South Africa using whole genome sequencing

Karlene Lambrechts, Pieter Gouws and Diane Rip*

Department of Food Science, Stellenbosch University, 7602, South Africa

* **Correspondence:** Email: dirip@sun.ac.za; Tel: +2721808 9739.

Supp;ementary

Table S1. Factory origin, lineage type, genes conferring sanitiser tolerance, and antibiotic susceptibility profiles of 46 *L. monocytogenes* isolates from seafood products, the FPE and clinical isolates in the Western Cape, South Africa.

Dates	Origin[a]	Description	Factory origin	*hly* gene	Lineage	*emrC* gene[b]	*bcrABC* gene[b]	Phenotypic antibiotic resistance[c]
**27/05/2019**	**Food**	Cold smoke trout ribbons	A	+	II			
**04/11/2019**	**Food**	Salmon ribbons	Seafood smokehouse	+	II			
**19/11/2021**	**Food**	Salmon ribbons		+	II			
**01/03/2019**	**Food**	Fish	B	+	I		+	
**01/03/2019**	**Food**	Fish	Seafood Processing and Manufacturing	+	I			
**29/05/2019**	**Food**	Fish		+	I	+		
**24/08/2022**	Food	Trout offcuts		+	II			
**31/08/2022**	Food	Trout slices		+	II		+	
**25/11/2021**	**Food**	Smoked trout roulades	C	+	I			TE, SXT
**25/11/2021**	**Food**	Smoked trout terrine	Condiment/RTE Food Manufacturer and Distributor	+	II		+	
**23/02/2019**	**Environment**	Fish room floor		+	II		+	
**11/08/2022**	**Environment**	Smoking room drain		+	I		+	
**14/09/2022**	Food	Salmon pate		+	II			
**05/09/2019**	**Food**	Salmon portion	D	+	II			
**30/08/2019**	Food	Sea bass fish fillet	Boutique retailer	+	II			
**05/09/2019**	**Food**	Oysters		+	II			
**28/10/2021**	Food	Cold smoke salmon	E	+	II			
**23/11/2021**	Food	Trout slices	Salmon Processing and Manufacturing	+	II			
**16/11/2021**	Food	Salmon fillet		+	II		+	
**26/10/2021**	Food	Trout offcuts		+	II			
**11/11/2021**	**Environment**	Salmon room drain		+	II		+	
**11/11/2021**	Environment	Drain offcuts		+	II			

Continued on next page

Dates	Origin[a]	Description	Factory origin	*hly* gene	Lineage	*emrC* gene[b]	*bcrABC* gene[b]	Phenotypic antibiotic resistance[c]
**17/11/2021**	Food	Salmon slices		+	II		+	SXT
**17/11/2021**	Food	Trout slices		+	II		+	
**23/11/2021**	Food	Trout strips		+	II		+	
**23/11/2021**	Food	Salmon strips		+	II		+	
**08/08/2021**	Food	Trout		+	II		+	
**20/07/2021**	Food	Trout	E	+	II		+	
**06/09/2021**	Food	Salmon	Salmon Processing and Manufacturing	+	II		+	
**23/09/2021**	Food	Tuna		+	II		+	
**08/07/2021**	Food	Trout		+	II		+	
**08/07/2021**	Food	Salmon		+	II		+	
**06/08/2021**	Food	Salmon		+	II		+	
**17/08/2021**	Environment	Smoker room Drain swab	F	+	II		+	
**17/09/2021**	Environment	Smoker room Drain swab	Meat Processing Facility (Various)	+	II		+	
**26/10/2018**	**Food**	Fresh hake	G	+	I			E
**26/10/2018**	**Food**	Fresh hake	Seafood Manufacturing Facility	+	I			C
**26/10/2018**	**Food**	Fresh hake		+	II			TE
**May 2019**	**Clinical**	Clinical 1	H	+	I			SXT
**Jun/Jul 2019**	**Clinical**	Clinical 2	NHLS	+	I			
**Jun/Jul 2019**	**Clinical**	Clinical 3		+	I			SXT
**Jun/Jul 2019**	**Clinical**	Clinical 4		+	I			SXT
**Jun/Jul 2019**	**Clinical**	Clinical 5		+	I			
**Aug/Sept 2019**	**Clinical**	Clinical 6		+	I			
**Aug/Sept 2019**	**Clinical**	Clinical 8		+	I			
**Dec 2019**	**Clinical**	Clinical 9		+	I			

[a]**Isolates in bold were selected for WGS**
[b] Sanitiser resistance genes information (by PCR) was not available at the point of selecting isolates for WGS
[c] **C**: Chloramphenicol; **E**: Erythromycin; **SXT**: Sulphamethoxazole/Trimethoprim; **TE**: Tetracycline

© 2024 the Author(s), licensee AIMS Press. This is an open access article distributed under the terms of the Creative Commons Attribution License (https://creativecommons.org/licenses/by/4.0)
